# Differential Regulation of Circulating Levels of Molecular Chaperones in Patients Undergoing Treatment for Periodontal Disease

**DOI:** 10.1371/journal.pone.0001198

**Published:** 2007-11-21

**Authors:** Alireza Shamaei-Tousi, Francesco D'Aiuto, Luigi Nibali, Andrew Steptoe, Anthony R. M. Coates, Mohamed Parkar, Nikos Donos, Brian Henderson

**Affiliations:** 1 Division of Microbial Diseases, UCL Eastman Dental Institute, University College London, London, United Kingdom; 2 St George's Hospital Medical School, London, United Kingdom; 3 Periodontology Unit, UCL Eastman Dental Institute and Hospital, University College London, London, United Kingdom; 4 Department of Epidemiology and Public Health, University College London, London, United Kingdom; Royal Hallamshire Hospital, United Kingdom

## Abstract

**Background:**

Evidence is emerging that molecular chaperones, in addition to their intracellular protein folding actions, can act as intercellular signaling proteins with an ability to modulate leukocyte function. Recent evidence has also shown that these proteins can exist in the circulation and may be involved in disease pathogenesis. We have used periodontitis and its treatment as a model of inflammation in the human to determine its effects on levels of circulating HSP10, HSP60 and BiP.

**Methodology/Principal Findings:**

A group of periodontal patients and matched controls were examined at the beginning of the study and then at 1 day and 6 months following periodontal or control therapy. Plasma levels of HSP10, HSP60 and BiP were measured by immunoassay and related to other plasma measures of inflammation. Periodontal patients had significantly less circulating levels of HSP10 or BiP compared with the controls. In contrast, more periodontal patients had intermediate levels of HSP60. Treatment of the periodontitis caused an increase in plasma levels of HSP10 although it had no effect on BiP. Treatment had no influence of HSP60 levels. Plasma HSP10 levels after therapy correlated with markers of periodontal clinical improvement.

**Conclusions/Significance:**

Circulating levels of molecular chaperones are influenced by local inflammation. HSP10 is known to be an anti-inflammatory factor. The marked decrease of this circulating protein in active inflammation and its recovery post-treatment suggests that it may have a role in controlling periodontal inflammation.

## Introduction

A major breakthrough in our understanding of cell biology was the discovery of the cell stress response and the role played in this response by inducible molecular chaperones [1], [2]. Molecular chaperones are proteins best known for their ability to fold unfolded proteins. However, two other, potentially related, biological functions of molecular chaperones were discovered contemporaneously with their folding actions. Many of the molecular chaperones of bacteria and parasites are potent immunogens [3], [4]. This has always been a paradoxical finding given the substantial sequence conservation between these proteins [4]. Although first discovered in the 1980s, it is only in the past decade that attention has focused on the finding that many molecular chaperones, particularly from eukaryotes, have intercellular signalling activity [5]. This may have remained just an interesting laboratory artefact but for the finding that a growing number of molecular chaperones exist in the human circulation. The first molecular chaperone (although it was not known that this was its function at the time) to be identified in human blood was heat shock protein (Hsp)10. This was identified as an immunosuppressive factor in the blood of pregnant women in the first trimester and was named early pregnancy factor (EPF) [6]. This was later identified as HSP10 [7]. Most attention has focused on circulating levels of the co-chaperone of HSP10, a protein termed HSP60. Circulating levels of HSP60 have been correlated with susceptibility to cardiovascular disease [8]–[13]. Members of the Hsp70 family such as Hsp70 [14], [15] and BiP [16] have been found in blood, and with the former, levels in the blood inversely correlate with cardiovascular disease severity [15].

Initial studies of the cell signalling actions of molecular chaperones suggested that they acted as pro-inflammatory mediators with actions similar to interleukin-1 or tumour necrosis factor alpha [5]. However, it has recently become apparent that certain molecular chaperones such as HSP10 [17] and BiP [18] act to inhibit the pro-inflammatory actions of myeloid cells. Indeed, HSP10 has recently been shown to inhibit joint inflammation in patients with rheumatoid arthritis [19].

Periodontal disease is a very common chronic inflammatory condition which can be simplistically viewed as a disease caused by the overgrowth of the normal oral bacterial microflora (dental plaque) associated with an exaggerated local host inflammatory response [20]. Treatment of this condition consists of mechanical removal of dental plaque and leads to rapid changes in inflammation locally, but can provide a systemic inflammatory insult due to the release of bacteria and their phlogistic products into the blood [21]. Our aim was therefore to utilize periodontitis and its treatment as a human model to determine how levels of circulating molecular chaperones relate to the presence and changes in local and systemic inflammation [22].

## Results

### Case-Control Study

The serum baseline characteristics of periodontal patients and controls are reported in Table 1. Age, gender, ethnicity and smoking habits were similar between groups. As expected from previous studies [21], the patients with periodontitis exhibited higher levels of CRP (median difference 0.6 mg/ml, 95%CI 0.1–1.2, P<0.05), IL-6 (median difference 0.6 pg/ml, 95%CI 0.3–1.0, P<0.01) and total leukocyte counts (mean difference 0.8 10^9^cells/L, 95%CI 0.2–1.4, P<0.01) when compared to controls. There was also an association between periodontitis and lower HDL levels (mean difference between groups 0.1 mmol/L, 95%CI 0–0.3, P<0.05) and higher glucose concentrations (mean difference between groups 0.3 mmol/L, 95%CI 0.1–0.6, P<0.05).

**Table 1:** Case control study

	Control Group (N = 48)	Periodontitis Group (N = 80)	P value*
Age, years	43 (40–47)	44 (43–45)	0.623
Male Gender, no. (%)	22 (46)	42 (50)	0.718
Smokers, no. Current (%)	10 (21)	25 (30)	0.309
Race or ethnic group, no. Caucasians(%)	39 (81)	57 (68)	0.108
CRP, mg/L	2.18 (1.07–3.28)	2.86 (2.10–3.62)	0.026
IL-6, pg/ml	2.17 (1.57–2.77)	2.34 (0.71–3.98)	0.005
Leucocytes count, 10^9^ cells/L	5.91 (5.50–6.32)	6.67 (6.11–7.23)	0.042
Cholesterol, mmol/L	5.34 (4.97–5.71)	4.82 (4.55–5.08)	0.148
HDL-Cholesterol, mmol/L	1.65 (1.52–1.77)	1.47 (1.36–1.59)	0.041
LDL-Cholesterol, mmol/L	3.02 (2.70–3.33)	2.76 (2.52–3.00)	0.264
Glucose, mmol/L	4.76 (4.55–4.97)	4.96 (4.81–5.11)	0.019
Triglycerides, mmol/L	1.42 (1.10–1.74)	1.26 (0.95–1.57)	0.182
HSP10, µg/ml; median(IQR)	549.50 (579.1)	0.10 (543.2)	<0.0001
BiP, µg/ml; median(IQR)	743.45 (1940.7)	478.75 (1225.8)	0.031
HSP-60, µg/ml; median(IQR)**	0.83 (1734.4)	32.50 (573.9)	0.478

* Differences were computed with parametric (Independent t-test) or non parametric (Mann-Whitney U-Test) dependent upon the data frequency distribution. Categorical variables were analyzed by Chi-square testing.

** See Figure 2.

**Figure 1:**
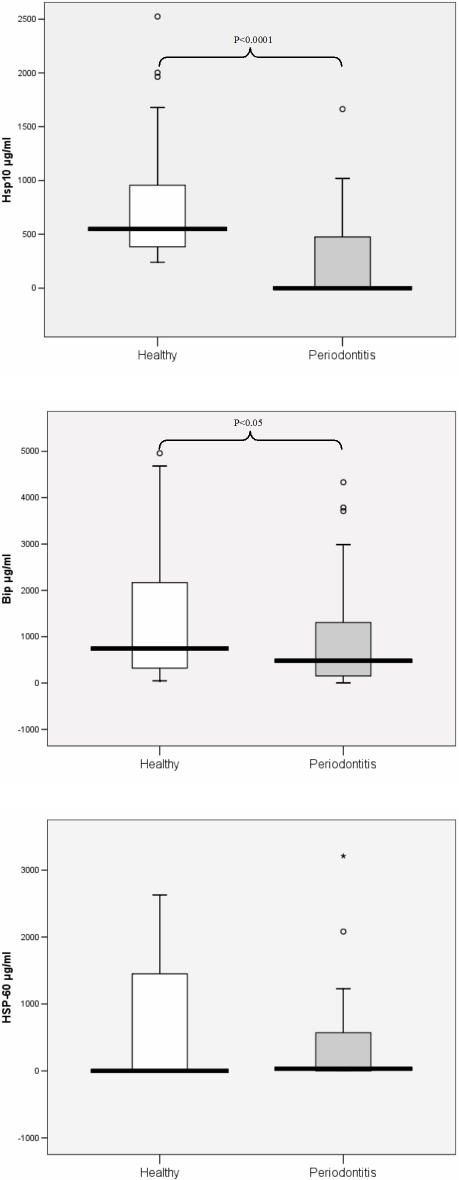
Differences in plasma concentrations between patients suffering from periodontitis (N = 80) and age-matched unaffected controls (N = 48). Boxes refer to the 25^th^ (bottom) and 75^th^ (up) percentiles and the median is the large horizontal line, fences refer to the 10^th^ (lower) and 90^th^ (upper) percentiles respectively. Open circles represent outliers whereas asterisks stand for extreme observations with the subject number. Statistical differences are computed with Mann-Whitney U-test.

Subjects with periodontitis had markedly lower plasma concentrations of HSP10 (median difference 404.8 µg/ml, 95% CI 332.4–499.1, P<0.0001) and lower concentrations of BiP (median difference 417.9 µg/ml, 95% CI 336.8–503.3, P<0.05) when compared to controls. After adjustment for age, gender, ethnicity and cigarette smoking, the odds for high HSP10 (≥1000 ng/ml) were 15.6 (95% CI 5.9–41.3) for controls compared to subjects with periodontitis. In contrast, there was a weaker association between periodontitis and HSP60 levels (Figure 1).

**Figure 2:**
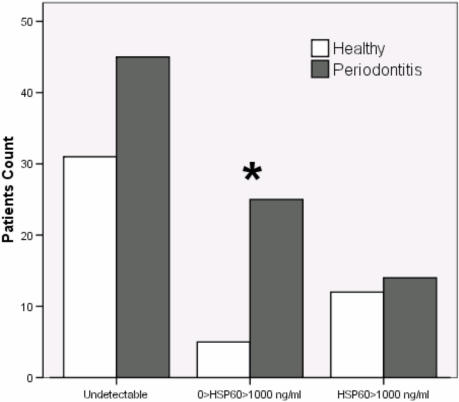
Number of individuals among test (periodontitis) and control (healthy) groups according to HSP60 levels being undetectable, less or greater than 1000 ng/ml. Asterisk refers to statistically significant differences (P<0.05) between groups within each category as computed with Chi-square test.

However, HSP60 levels were stratified into those with: undetectable, between 1 and 1,000 ng/ml and >1,000 ng/ml HSP60 and we found a significant difference between controls and patients in the numbers of individuals having medium (between 1 and 1,000ng/ml) levels of circulating HSP60. Thus many more periodontal patients had medium levels of circulating HSP60 and the odds for medium HSP60 were 3.4 (95% CI 1.2–10) higher for patients with periodontitis compared to controls (Figure 2).

We also observed a consistent inverse association between plasma concentrations of HSP10and clinical periodontal parameters (averaged whole mouth probing depths, r = 0.31, P = 0.022; averaged whole mouth number of periodontal lesions r = 0.32, P = 0.032 and whole mouth supra-gingival plaque scores, r = 0.33, P = 0.002).

### Intervention Study

**Figure 3:**
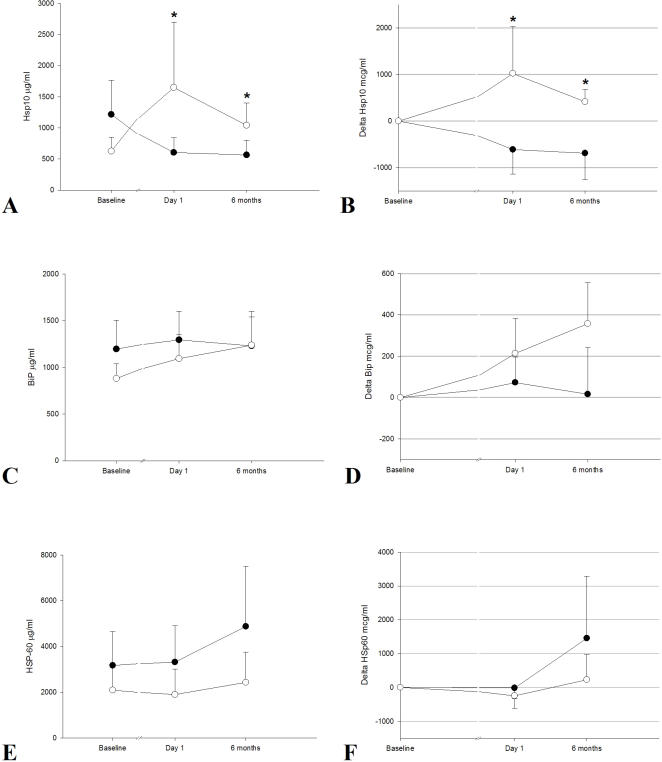
Mean values (standard errors) and mean changes (standard errors) (compared to baseline) of HSP10 (A–B), BiP (C–D) and HSP60 (E–F) before, 24 hrs after and 6 months after periodontal therapy. Positive differences indicate a relative increase in plasma concentrations of various markers compared to the pre-treatment baseline. Subjects who received intensive periodontal therapy (open circles, N = 40) showed greater plasma concentrations of HSP10 at each time visit when compared to control therapy group subjects (filled circles, N = 40). No other changes were observed. Asterisks refer to statistically significant difference (P<0.05) between groups as computed with analysis of covariance.

After 6 months of therapy, subjects in the IPT group presented with lower gingival plaque scores (mean between groups difference 29%, 95% 21–37, P<0.0001) and lower numbers of periodontal pockets (mean between group difference 62, 95% 53–71, P<0.0001) when compared to controls. Of the circulating molecular chaperones being measured, we only observed an association between periodontal therapy and the concentrations of plasma HSP10 (Figure 3). Within 24hrs of therapy the IPT patients exhibited substantial increases in their plasma concentrations of HSP10 (difference between changes from baseline between groups 1633 ng/ml , 95%CI 181–3086, P = 0.21) but with great variability among individuals. This increase in circulating HSP10 was still measurable 6 months after therapy (difference between changes from baseline between groups 1105, 95%CI 89–2575, P = 0.034) (Figure 3). The covariance analysis revealed that these changes were independent of age, gender, ethnicity and smoking differences between study treatment groups. There was also a dose-response positive association between improvements in clinical periodontal parameters (reduction in number of periodontal pockets lesions between baseline and 6 months) and increases in HSP10 plasma concentrations (r = 0.29, P = 0.028). No other differences were observed in all the other biological markers between study groups after 6 months of therapy including the significant difference in the proportion of patients with HSP60 levels between 1 and 1,000 ng/ml.

## Discussion

The inflammatory response is a complex system involving the interactions of leukocytes with activated vascular endothelial cells. This system has to be tightly controlled, and the discovery of cytokines, beginning in the 1970s, provided what appeared to be the major class of inflammatory control protein [23]. Inflammation is stressful, and it is perhaps not surprising that it is associated with alterations in heat shock/cell stress protein expression [24], [25]. Two unexpected discoveries, which are still the cause of some controversy, are that: (i) many molecular chaperones have intercellular signaling actions particularly with myeloid, lymphoid and vascular endothelial cells [26] and (ii) molecular chaperones can be found in the blood [27]. Initial studies suggested that molecular chaperones were pro-inflammatory factors able to activate myeloid and vascular endothelial cells [28]. However, subsequent work has revealed that molecular chaperones such as BiP [18] and HSP10 [17] are able to inhibit myeloid cell activation. It has also been shown that human HSP60, while able to activate human myeloid cells [reviewed in 28] has the ability to inhibit lymphocyte activation [29]. The first molecular chaperone to be identified in blood from normal individuals was HSP60 [30]. Since then levels of this molecular chaperone have been shown to correlate with measures of cardiovascular pathology in a number of studies [27]. However, almost nothing is known about circulating levels of other molecular chaperones such as BiP or HSP10 in human blood or how such levels relate to disease status.

Periodontal disease is a very common condition in which inflammation of the gingivae is associated with destruction of the periodontal ligament and alveolar bone which support the dentition. This disease is a response to overgrowth and downgrowth of suspected periodontal bacterial pathogens. Non-surgical and surgical periodontal therapy results in removal of these bacteria which can rapidly decrease the local inflammation in the gingivae while causing a short-term systemic inflammatory response as a result of local tissue damage and the release of bacterial phlogistic mediators such as Gram-negative lipopolysaccharide [21]. This ability to be able to modulate the human inflammatory response makes periodontal disease a potential model system for studying the control of inflammation directly in the human subject.

In the present study we have assayed the concentrations of three molecular chaperones, HSP10, HSP60 and BiP in the blood of patients with periodontal disease and compared this with a group of age-, gender- and ethnically-matched non-diseased controls. This revealed that there was no difference in the overall concentrations of HSP60 in both groups. However, HSP60 levels in blood are stratified with around 50% having no measurable protein, 25% having high levels (>1 µg/ml) and the remainder with levels in the range 1–1,000 ng/ml. Comparison of these three distinct concentration bands revealed a significantly higher number of periodontal patients with intermediate levels of HSP60 compared to controls. Evidence from the literature tends to suggest that high levels of HSP60 are pro-inflammatory but lower levels are anti-inflammatory [29]. It is interesting to speculate whether individuals with low levels of HSP60 have their local anti-bacterial defences compromised by the action of this protein?

Levels of the endoplasmic reticulum cell stress protein BiP were significantly lower in the plasma of the periodontal patients than in controls. This protein is an active anti-inflammatory molecule both *in vitro* and *in vivo,* and so lower levels in individuals with on-going inflammation are a consistent finding.

The results for HSP10 levels were even more striking with the concentrations of this stress protein in the periodontal patients being very low compared to the matched group of controls. Of interest was the finding in the periodontitis patients that there was a consistent inverse association between HSP10 plasma concentrations and clinical parameters of periodontal disease severity, such as the number of periodontal pockets or dental plaque scores. The very low levels of HSP10 in the periodontal patients could have been due to genetic or environmental factors. Fortunately, the intervention protocol, which acted to remove the inflammatory stimuli in these individuals, gave us a chance to test if plasma levels of molecular chaperones were stable (genetic) or were environmentally controlled.

Periodontal patients were randomly divided into two groups. The control group received a standard cycle of supragingival mechanical scaling and polishing. The intensive treatment group underwent a prolonged session of full-mouth removal of subgingival dental plaque biofilms under local analgesia as described [31]. The latter therapy releases bacterial pro-inflammatory molecules into the bloodstream and causes an acute phase response with endothelial dysfunction [31]. This is associated with elevations of C-reactive protein and the pro-inflammatory cytokine, interleukin (IL)-6 [31], [32]. In spite of the elevation in the levels of these pro-inflammatory indicators, the levels of HSP10 in the circulation were increased significantly at one day following therapy and were still elevated after 6 months, when the acute phase protein and endothelial dysfunction had returned to baseline, showing that levels of this protein are environmentally controlled. In contrast, there were no significant changes in the levels of HSP60 or BiP after therapy.

Patients with moderate to severe periodontitis reveal significant lowering of the plasma levels of two molecular chaperones which have anti-inflammatory actions–HSP10 and BiP when compared with controls. Both of these proteins are able to inhibit the activation of macrophages [17], [33]. BiP has been shown to block experimental collagen-induced arthritis in the mouse [18] and is in early phase clinical trials for the treatment of rheumatoid arthritis. HSP10, or early pregnancy factor (EPF) inhibits the development of autoimmune disease in rats [34] and has recently been administered to a small number of patients with rheumatoid arthritis with therapeutic benefit [35]. The mechanism of action of BiP, at least in inbred mice, is the induction of regulatory lymphocytes that are largely controlled by IL-4 [18]. The mechanism of immunosuppression of HSP10 is less well defined but is believed to inhibit intracellular signaling pathways stimulated by ligand interaction with selected Toll-like receptors (TLRs) [17]. It is not known if this effect is mediated at the cell surface or is an intracellular effect. There is insufficient evidence to understand the exact role of these molecules at the gingival levels.

The decreased circulating concentrations of HSP10 in patients with periodontitis could either be due to decreased synthesis, with this protein therefore acting like serum albumin and transferrin as a negative acute phase reactant, or to removal from the circulation, as part of the anti-inflammatory activity of this protein. Decreased synthesis of HSP10 is unlikely as the genes encoding both HSP10 and HSP60 are organized in a head-to-head fashion with a shared bidirectional promoter [35]. If HSP10 synthesis did decrease in periodontal disease a corresponding decrease in HSP60 concentrations would be expected, and was not observed in this study. Moreover, unlike conventional acute phase proteins that are largely synthesized in the liver, HSP10 and HSP60 are found in every cell as nuclear encoded genes which function within the mitochondria. Thus the reduction in HSP10 levels in patients with periodontitis must be due to the removal of this protein from the blood, potentially as part of a natural anti-inflammatory mechanism. Treatment of the gingival/periodontal inflammation, which results in local dampening of the inflammatory mechanisms, would therefore be expected to stop the removal of this protein from the circulation and increase levels in the blood. What is surprising is how rapidly levels increased following an intensive session of periodontal therapy.

In conclusion, we have discovered that periodontal disease is associated with significant decreases in the circulating concentrations of one endoplasmic reticulum molecular chaperone, BiP which is a key controller of the unfolded protein response [36] and a mitochondrial molecular chaperone involved in protein folding within this organelle. Treatment of the gingival inflammation results in a return of HSP10 levels to those found in controls but has no effect on the lowered levels of BiP. These finding reveal that circulating molecular chaperones are important indicators of disease status and response to therapy and argues that we need to understand more about the levels of these proteins in blood and what controls their concentrations.

## Materials and Methods

### Case-Control Study

Data for this experiment derives from a case-control investigation including all individuals recruited among the population referred for care to the Eastman Dental Hospital. Eighty subjects with a diagnosis of moderate to severe periodontitis [37] were enrolled at the Periodontology Unit, and 48 controls were selected among patients attending other units (Oral Surgery or Restorative Clinics) and presenting without clinical and radiographic signs of periodontitis. All individuals were: (i) free from systemic diseases as assessed by the examining clinician; (ii) not pregnant if females and; (iii) not on any medication. In addition, control subjects were included only if they did not report a history of tooth loss due to periodontitis. All subjects gave written informed consent and the study protocol had been reviewed and approved by University College London Hospitals and Eastman Dental Hospital joint ethics committee.

Control subjects received a basic clinical examination (basic periodontal screening score (BPE)), whereas a cluster of periodontal parameters were collected from patients suffering from periodontitis. These included gingival probing depth (PPD) and recession of the gingival margin (GR) relative to the cemento-enamel junction at 6 sites per tooth. Presence or absence of supra-gingival dental plaque and gingival bleeding on probing were also recorded. Averaged whole mouth number of periodontal lesions (probing depth >4 mm), full mouth gingival bleeding scores (relative number of sites with gingival bleeding on probing) and full mouth plaque scores (relative number of sites with detectable plaque) were recorded for each individual [38].

### Intervention Study

For this experiment we used data from a clinical intervention trial of patients with severe periodontitis. Inclusion criteria and clinical parameters recorded for this population were as described above. Eighty individuals included in the study received basic oral hygiene instructions and then were randomized (permuted block approach) to an intensive (IPT, N = 40) or standard cycle of periodontal therapy (SPT, N = 40). Allocation to treatment was concealed in opaque envelopes which were opened at the time of therapy. SPT consisted of a session of supra-gingival scaling and polishing of the whole dentition. In contrast, the IPT group subjects received sub-gingival mechanical instrumentation under local anaesthesia. All patients were re-examined 24 hrs and 6 months after therapy. This is because we previously showed that intensive periodontal therapy causes a transient systemic inflammatory response (peak 1 day after therapy) resolving only after 6 months [21], [22]. No adverse events were reported.

### Laboratory Analyses

Blood samples were collected at the baseline visit for both investigations and also after therapy (24 hrs and 6 months) in the intervention study. Plasma samples were immediately processed and stored at −70°C until the analyses were performed. Routine markers (full blood count, lipids and glucose levels) were determined using standard biochemistry procedures. Serum inflammatory markers were determined by high sensitivity immunoassays (C-reactive protein: Cobas Integra 700, Roche, Mannheim, Germany; IL-6: Quantikine HS, R&D System, Minneapolis).

Two site ELISAs were used to measure the concentrations of HSP10, HSP60 and BiP in plasma samples. The assay for HSP60 was as described [10], [11]. To assay BiP, ELISA plates (Immunosorp, Nunc) were coated with 0.125 µg mouse anti-BiP (BD Transduction labs) in PBS overnight at 4°C. Non-specific binding was blocked by the addition of 0.1% BSA in PBS 0.05% Tween-20 serum (Sigma) for 1 hour at room temperature. After washing, recombinant BiP (0 to 10 000 ng/mL) or dilutions of human plasma were added, and plates were incubated for two hours at 37°C and then washed. Samples were incubated with rabbit anti-BiP polyclonal IgG (Santa Cruz Biotechnology; 1:400 dilution in PBS 0.05% Tween-20 supplemented with 4% filtered normal mouse serum) for 2 hours 37°C the wells. After washing, biotinylated anti-rabbit IgG (Santa Cruz Biotechnology; 1:10, 000 dilution) was added and incubated at 37°C for one hour. After a further wa step, samples were incubated with streptavidin HRP (Sigma; 1∶2 000 dilution) for 45 minutes at 37°C. Binding of conjugated antibody was detected using TMB substrate. The reaction was stopped with 1 mol/L H_2_SO_4_, and absorbance was determined at 492 nm using a Dynex plate reader. Each plasma sample was assayed in triplicate. Human plasma HSP10 was measured using a newly developed two-site ELISA. ELISA plates (Immunosorp, Nunc ) were coated with 10 ng of goat polyclonal anti-HSP10 antibody (clone FL-102, corresponds to the full length of human HSP10, Santa Cruz Biotechnology) in PBS overnight at 4°C. Plates were washed in wash buffer (0.5 mol/L NaCl, 2,5 mmol/L NaH_2_PO_4_, 7,5 mmol/L, Na_2_ HPO_4_ and 0.1% Tween-20), and nonspecific binding sites were blocked by incubation with 1% BSA (Sigma) in wash buffer for 4 hours at 37°C. After washing, recombinant HSP10 (Stressgen; 0 to 10, 000 ng/ml) or dilutions of human plasma were added, and plates were incubated overnight at 4°C and then washed. Bound HSP10 was detected by incubation with goat polyclonal antibody to HSP10 (clone C-20, Santa Cruz Biotechnology) at 1∶500 for 1 hour at 37°C. After washing, plates were incubated with HRP conjugated anti goat IgG (Sigma; 1∶5000) for 1 hour at 37°C. Binding of conjugated antibody was detected using Sigma fast OPD (1,2-phenylenediamine dihydrochloride) substrate. The reaction was stopped by 1 mol/L H_2_SO_4_, and absorbance was determined at 492 nm using a Dynex plate reader. Each plasma samples was assayed in triplicate. Plasma from individuals with the highest plasma levels of HSP10 were assayed at least twice in separate assays.

### Statistical Analysis

As there were no previous report on the association between periodontitis and molecular chaperones, no formal sample size calculations were available. Data are reported as means and 95% confidence intervals unless otherwise specified. In the case-control experiment we compared differences in all variables between groups by parametric (ANOVA) or non parametric (Mann-Whitney U-test) statistics depending on the shape of the distribution's curve of the data. Differences between categorical variables were computed using Chi-square testing. Logistic regression models were used to ascertain differences between cases and control or low versus high (≥1000 ng/ml) plasma concentrations of HSP10, HSP60 and BiP. Differences between treatment groups for each marker in the intervention trial were computed using analysis of covariance. Logarithmic transformation of the data was performed where appropriate. Age, gender, ethnicity and cigarette smoking were included in each model as covariates. Non parametric correlation analyses (Spearman rank test) were performed to evaluate the association between bio-markers and clinical periodontal parameters (before and after therapy). A two-sided value of p<0.05 was considered statistically significant using SPSS ver.13.
